# Clinicopathological characteristics and survival outcomes in adenosquamous carcinoma of the lung: a population-based study from the SEER database

**DOI:** 10.18632/oncotarget.23550

**Published:** 2017-12-21

**Authors:** Jian Wang, Bi Lian, Ling Ye, Jie Hu, Yuanlin Song

**Affiliations:** ^1^ Department of Pulmonary Medicine, Zhongshan Hospital, Fudan University, Shanghai 200030, China; ^2^ Department of Breast Surgery, Key Laboratory of Breast Cancer in Shanghai, Fudan University Shanghai Cancer Center, Fudan University, Shanghai 200030, China

**Keywords:** adenosquamous carcinoma, adenocarcinoma, squamous cell carcinoma, prognosis

## Abstract

Adenosquamous carcinoma (ASC) of the lung is an unusual histology type in non-small-cell lung cancers. Due to its rarity, the clinicopathological characteristics and survival outcomes of the lung ASC are incompletely understood. We used the Surveillance, Epidemiology, and End Results (SEER) database to enroll 203,208 eligible patients, including 4,245 ASC, 124,253 adenocarcinoma (ADC) and 74,710 squamous cell carcinoma (SCC) patients. To date, this is the largest cohort in a study for ASC of the lung. With regard to age, sex, race, year of diagnosis, tumor size and SEER stage, ASC was intermediate between ADC and SCC. However, compared with ADC and SCC patients, ASC patients presented with a higher tumor grade and lower prevalence of nodal metastasis. More ASC patients underwent surgery and a lower proportion underwent radiation treatment and chemotherapy. Kaplan-Meier analysis showed that ASC patients had a better prognosis than ADC and SCC patients, but stratified analysis showed that the prognosis of ASC patients was worse than that of ADC and SCC patients in surgery and non-surgery subgroup. Multivariate analysis further confirmed that the ASC histology type was a risk factor for poor prognosis with respect to ADC and SCC. Using the propensity score matching to 1:1 match ASC with ADC or SCC, we found that ASC patients had worse survival than ADC and SCC patients. Subgroup analysis further demonstrated that ASC was a more aggressive histology type with a worse prognosis. These results provided a deep understanding of ASC, which contributed to better clinical diagnosis and treatment.

## INTRODUCTION

Adenosquamous carcinoma (ASC) of the lung is defined, according to World Health Organization (WHO), as a tumor having ≥ 10% components of both adenocarcinoma (ADC) and squamous cell carcinoma (SCC) [1, 2]. Compared with ADC or SCC, ASC is a relatively rare histology type of non-small cell lung cancers, because the prevalence of ASC has been estimated to be 0.4–4% of all lung cancers [3]. Due to its rarity, the clinicopathological characteristics and survival outcomes of ASC have not been clarified.

Differential demographic and clinical characteristics of ASC were found in different studies as for the limited case numbers. Mordant et al. [4] reported that ASC patients presented with similar features to those of ADC patients with regard to age, sex and smoking history, but had larger tumor size compared with ADC and SCC cases. However, Maeda et al. [5] showed that ASC patients were younger than ADC and SCC cases, and were intermediate between ADC and SCC patients with regard to sex. The tumor size of ASC patients was similar to that of SCC patients. Most studies have suggested that ASC patients have worse survival compared with ADC and SCC cases due to its more aggressive biologic behaviors, but definitive conclusions on the prognosis have not been drawn. Filosso et al. [6] reported that the 3- and 5-year survival of ASC patients after surgery were 25% and 15%, respectively, and overall survival was worse than that for ADC and SCC cases. Gawrychowski et al. [7] showed that the overall survival at 5 and 10 years in ASC patients after surgery were 25.4% and 19.2%, respectively, and that ASC carried a worse prognosis compared with ADC and SSC. Cooke et al. [8] identified 872 ASC patients who underwent surgery from the Surveillance, Epidemiology, and End Results (SEER) database (1998–2002), and found that ASC had a worse prognosis than ADC and SCC. However, some studies have not showed a significant difference in the prognosis between ASC and ADC or SCC [0]. In 1999, WHO reclassified histology criteria for lung ASC [1], but most cases in these studies were obtained before 1999, which resulted in confounding of the clinicopathological characteristics and survival outcomes of ASC.

So far, few scholars have attempted to identify the prognostic factors for ASC. Watanabe et al. [11] enrolled 53 ASC patients and found that tumor size > 5 cm and peritumoral inflammatory changes on computed tomography (CT) were independent prognostic factors for ASC. Filosso et al. [6] reviewed of 48 ASC patients retrospectively, and identified that distant metastasis, tumor perineural invasion and tumor stage were risk factors for prognosis and that receiving adjuvant chemotherapy was a protective factor for the prognosis in ASC patients. The small number of ASC patients in these studies reduced the reliability and practical importance of their results for the clinical diagnosis and treatment of ASC. A large population-based and multi-center study to clarify the clinicopathological characteristics and survival outcomes of ASC is lacking.

In the present study, we obtained the clinicopathological and prognostic data of patients with ASC, ADC and SCC from the SEER (2000–2014), which was a large population-based database supported by the American National Cancer Institute. Differences between ASC and ADC or SCC were identified to make a deeper understand on ASC.

## MATERIALS AND METHODS

### Ethics statement

We have signed the SEER Research Data Agreement for the access to the SEER data using the reference number 11782-Nov2016. The informed consent was not required in this study because there was no personal identifying information in SEER database. This study was approved by the Ethical Committee and Institutional Review Board of Zhongshan Hospital, Fudan University.

### Study population

We obtained patient data from the SEER database (Submission, November 2016). It contains research data from 18 population-based cancer registries and the Hurricane Katrina impacted Louisiana cases from 1973 to 2014, and covers approximately 27.8% of the American population. Eligible patients were selected based on the tool SEER^*^Stat v8.3.4 (released 23 March 2017) from the National Cancer Institute.

Patients diagnosed based on histology with lung ASC, ADC or SCC were enrolled in the present study. Inclusion criteria were shown as follows: age at the diagnosis ≥ 18 years; site recode ICD-O-3/WHO 2008 (International Classification of Diseases for Oncology, Third Edition) was restricted to “Lung and Bronchus”; pathologically confirmed ASC (ICD-O-3 8560/3) was selected whereas the pathologic type of ADC and SCC were identified according to the criteria stated in the previous study [12]; the diagnosis was not confirmed by autopsy or death certificate; only one malignant primary tumor was diagnosed. We excluded patients who had incomplete survival data. Patients diagnosed before 2000 were also excluded because WHO updated the definition of lung ASC in 1999.

### Covariates

The covariates including age, race, sex, year of diagnosis, marital status, tumor grade, tumor size, SEER stage, nodal status, surgery, radiation and chemotherapy were extracted for further analysis. We classified age into four groups: < 60, 60–69, 70–79, and ≥ 80. The year of diagnosis was classified into three groups: 2000–2004, 2005–2009, and 2010–2014. Tumor size (cm) was categorized as follows: ≤ 3, > 3 and ≤ 5, > 5 and ≤ 7, and > 7. As for the different criteria for American Joint Committee on Cancer (AJCC) stage in the SEER database during the study period, the SEER stage was used to describe the tumor stage. It was classified into localized, regional and distant according to the SEER program.

Cancer-specific survival (CSS) was used as the primary outcome of our study. CSS was defined as the follow-up time from the diagnosis to death due to lung cancer. The cutoff date for follow-up was 31 December 2014. Any patient who died due to other causes before this cutoff date or who was alive on the date of last contact was censored.

### Statistical analysis

The demographic and clinical characteristics of ASC patients were compared with those of ADC or SCC patients using the chi-square test. The Kaplan-Meier method was used to generate survival curves. Differences between these curves were analyzed by the log-rank test. After proportional hazards assumption was checked using Schoenfeld residuals, univariate and multivariate Cox proportional models were used to identify prognostic factors and the results were shown as hazard ratios (HRs) and 95% confidence intervals (95% CI). To reduce the effects of differences in characteristics among the three groups on CSS, a propensity score matching (PSM) method to 1:1 match ASC with ADC or SCC patients, respectively, was used. The factors for matching ASC with ADC were age, race, sex, year of diagnosis, tumor grade, tumor size, SEER stage, nodal status, surgery, radiation and chemotherapy, while the factors for matching ASC with SCC were age, race, sex, year of diagnosis, marital status, tumor grade, tumor size, SEER stage, nodal status, surgery, and radiation. PSM was undertaken using the psmatch2 module in Stata v14.0 (StataCorp, College Station, TX, USA). Subgroups analysis was done using a multivariate Cox proportional model to determine the HRs of ASC *vs.* ADC or ASC *vs.* SCC in a matched population according to covariate stratification. Grade I subgroup was excluded because the number of patients was small.

All statistical analysis were carried out using SPSS v20.0 (IBM, Armonk, NY, USA). *P* < 0.05 (two-sided) was considered significant.

## RESULTS

### Baseline characteristics of the study cohort

The demographic and clinical characteristics of the three patient groups were shown in Table 1. A total of 203,208 eligible patients were identified: 4,245 with ASC, 124,253 with ADC and 74,710 with SCC. A significant difference between ASC and ADC cases was found with respect to age, race, sex, year of diagnosis, tumor grade, tumor size, SEER stage, nodal status, surgery and chemotherapy. Compared with ADC patients, ASC patients were older (70–79: 33.3% *vs.* 28.4%; ≥ 80: 13.3% *vs.* 12.7%; *P* < 0.001), were predominantly male (55.3% *vs.* 49.0%; *P* < 0.001), had more white race (82.0% *vs.* 79.4%; *P* < 0.001), had a higher tumor grade (III-IV: 49.0% *vs.* 30.9%; *P* < 0.001), had greater tumor size (5 cm < size ≤ 7 cm: 14.8% *vs.* 11.0%; size > 7 cm: 8.4% *vs.*7.0%; *P* < 0.001) and a higher proportion of ASC patients underwent surgery (50.4% *vs.* 32.5%; *P* < 0.001). However, ASC patients had the lower prevalence of distant metastasis according to SEER stage (38.5% *vs.* 53.2%; *P* < 0.001) and nodal metastasis (50.6% *vs.* 51.0%; *P* < 0.001), and fewer ASC patients underwent chemotherapy (42.9% *vs.* 47.9%; *P* < 0.001). There was no significant difference between ASC and ADC cases with regard to marital status and radiation treatment. When comparing ASC patients with SCC cases, a significant difference was found in age, race, sex, year of diagnosis, marital status, tumor grade, tumor size, SEER stage, nodal status, surgery, and radiation, but not for chemotherapy. Compared with SCC cases, ASC patients were younger (< 60: 23.0% *vs.* 18.5%; *P* < 0.001), had a higher tumor grade (III-IV: 49.0% *vs.* 38.3%; *P* < 0.001) and had the higher prevalence of distant metastasis according to SEER stage (38.5% *vs.* 37.7%; *P* < 0.001). More ASC patients were married (54.8% *vs.* 51.4%; *P* < 0.001) and underwent surgery (50.4% *vs.* 29.1%; *P* < 0.001). However, compared with SCC cases, fewer ASC patients were male (55.3% *vs.* 64.1%; *P* < 0.001), had white race (82.0% *vs.* 82.2%; *P* < 0.001) and underwent radiation treatment (40.3% *vs.* 47.9%; *P* < 0.001). They had a smaller tumor size (size ≤ 3 cm: 35.0% *vs.* 23.5%; *P* < 0.001) and the lower prevalence of nodal metastasis (50.6% *vs.* 51.5%; *P* < 0.001), compared with SCC patients. In summary, compared with ADC and SCC patients, ASC patients had a higher tumor grade and lower prevalence of nodal metastasis. More ASC patients underwent surgery and fewer had radiation treatment and chemotherapy. However, ASC cases were intermediate between ADC and SCC patients with regard to age, race, sex, year of diagnosis, tumor size and SEER stage.

**Table 1:** The demographic and clinical characteristics of these three patient groups

Characteristics	ASC *N* = 4245 (%)	ADC *N* = 124253 (%)	SCC *N* = 74710 (%)	*p* value^a^ ASC vs. ADC	*p* value ASC vs. SCC
**Age**				< 0.001	< 0.001
< 60	977 (23.0)	34609 (27.9)	13814 (18.5)		
60–69	1292 (30.4)	38618 (31.1)	23782 (31.8)		
70–79	1413 (33.3)	35259 (28.4)	26064 (34.9)		
≥ 80	563 (13.3)	15767 (12.7)	11050 (14.8)		
**Sex**				< 0.001	< 0.001
Female	1896 (44.7)	63397 (51.0)	26802 (35.9)		
Male	2349 (55.3)	60856 (49.0)	47908 (64.1)		
**Race**				< 0.001	< 0.001
White	3481 (82.0)	98621 (79.4)	61441 (82.2)		
Black	471 (11.1)	14552 (11.7)	9390 (12.6)		
Others^b^	285 (6.7)	10757 (8.7)	3732 (5.0)		
Unknown	8 (0.2)	323 (0.3)	147 (0.2)		
**Year of diagnosis**				< 0.001	0.006
2000–2004	1192 (28.1)	31943 (25.7)	22298 (29.8)		
2005–2009	1352 (31.8)	39098 (31.5)	24202 (32.4)		
2010–2014	1701 (40.1)	53212 (42.8)	28210 (37.8)		
**Marital status**				0.39	< 0.001
Married	2327 (54.8)	67271 (54.1)	38385 (51.4)		
Not married^c^	1730 (40.8)	51803 (41.7)	33219 (44.5)		
Unknown	188 (4.4)	5179 (4.2)	3106 (4.2)		
**Grade**^d^				< 0.001	< 0.001
I	42 (1.0)	9567 (7.7)	1819 (2.4)		
II	907 (21.4)	30575 (24.6)	22651 (30.3)		
III-IV	2079 (49.0)	38381 (30.9)	28601 (38.3)		
Unknown	1217 (28.7)	45730 (36.8)	21639 (29.0)		
**Tumor size (cm)**				< 0.001	< 0.001
≤ 3	1486 (35.0)	46030 (37.0)	17593 (23.5)		
3–5	1164 (27.4)	29313 (23.6)	19050 (25.5)		
5–7	627 (14.8)	13619 (11.0)	12877 (17.2)		
> 7	355 (8.4)	8650 (7.0)	9860 (13.2)		
Unknown	613 (14.4)	26641 (21.4)	15330 (20.5)		
**SEER stage**				< 0.001	< 0.001
Localized	985 (23.2)	25319 (20.4)	16713 (22.4)		
Regional	1572 (37.0)	30518 (24.6)	27878 (37.3)		
Distant	1634 (38.5)	66050 (53.2)	28146 (37.7)		
Unknown	54 (1.3)	2366 (1.9)	1973 (2.6)		
**Nodal status**				< 0.001	< 0.001
No	1861 (43.8)	48591 (39.1)	29857 (40.0)		
Yes	2146 (50.6)	63330 (51.0)	38470 (51.5)		
Unknown	238 (5.6)	12332 (9.9)	6383 (8.5)		
**Surgery**				< 0.001	< 0.001
No	2091 (49.3)	83330 (67.1)	52414 (70.2)		
Yes	2138 (50.4)	40349 (32.5)	21766 (29.1)		
Unknown	16 (0.4)	574 (0.5)	530 (0.7)		
**Radiation**				0.085	< 0.001
No/Unknown	2592 (61.1)	74229 (59.7)	38891 (52.1)		
Yes	1653 (38.9)	50024 (40.3)	35819 (47.9)		
**Chemotherapy**				< 0.001	0.26
No/Unknown	2424 (57.1)	64712 (52.1)	42003 (56.2)		
Yes	1821 (42.9)	59541 (47.9)	32707 (43.8)		

Abbreviations: ASC, adenosquamous carcinoma; ADC, adenocarcinoma; SCC, squamous cell carcinoma; SEER, Surveillance Epidemiology and End Results database.

^a^*p* value between ASC and ADC or SCC was calculated by chi-square test, respectively.

^b^Others included American Indian/Alaskan native, and Asian/Pacific islander.

^c^Not married included separated, single (never married), divorced, unmarried or domestic partner and widowed.

^d^Grade I is well-differentiated; Grade II is moderately differentiated; Grade III is poorly differentiated; Grade IV is undifferentiated.

### Comparison survival among the three groups

**Figure 1:**
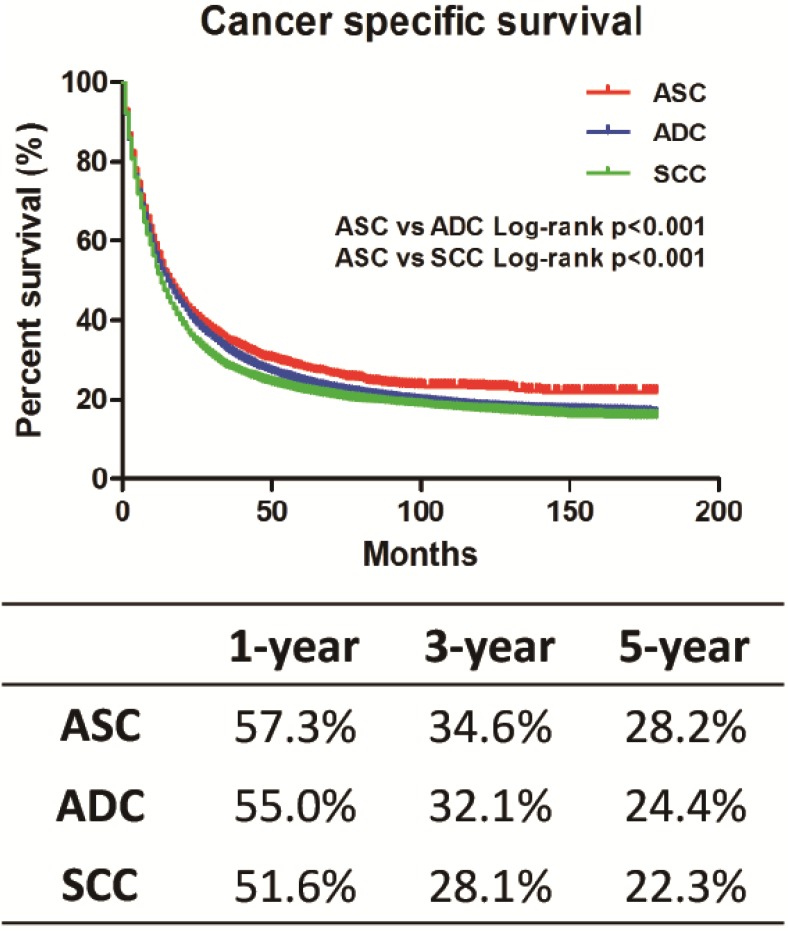
Kaplan-Meier plot and log-rank test for the cancer-specific survival (CSS) among these three histological types ASC vs. ADC, *p* < 0.001; ASC vs. SCC, *p* < 0.001.

The Kaplan-Meier method was used to analyze CSS among these three histology types (Figure 1). Unexpectedly, ASC patients carried a better prognosis than ADC and SCC patients, respectively (ASC *vs.* ADC, *P* < 0.001; ASC *vs.* SCC, *P* < 0.001). CSS at 1, 3 and 5 years for ASC patients were 57.3%, 34.6% and 28.2%, respectively, and all of these CSS values were higher than those of ADC and SCC patients. The SCC patients seemed to have the worst survival. Univariate and multivariate Cox proportional hazard models were used to identify the prognostic factors associated with CSS (Table 2). In the univariate analysis, all of the covariates (histology type, age, race, sex, year of diagnosis, marital status, tumor grade, tumor size, SEER stage, nodal status, surgery, radiation and chemotherapy) showed a significant association with CSS. The multivariate analysis adjusted for all of these covariates, and found that all of these covariates remained prognostic factors for CSS. Interestingly, the ASC histology type (set as the reference) was found to be a protective factor compared with ADC or SCC in the univariate analysis. However, the multivariate analysis showed that ASC patients had worse CSS than ADC or SCC patients. Similarly, chemotherapy was a risk factor for CSS in the univariate analysis but was a protective factor for CSS in the multivariate analysis. Besides, SEER stage and surgery were considered to be the most significant prognostic factors for CSS.

**Table 2:** Univariate and multivariate analysis of cancer-specific survival (CSS) among these three groups

Characteristics	Univariate analysis		Multivariate analysis	
	HR (95% CI)	*p* value	HR (95% CI)	*p* value
**Histological type**				
ASC	Reference	—	Reference	—
ADC	1.09 (1.05–1.13)	< 0.001	0.87 (0.84–0.90)	< 0.001
SCC	1.18 (1.13–1.22)	< 0.001	0.88 (0.84–0.91)	< 0.001
**Age**				
< 60	Reference	—	Reference	—
60–69	0.97 (0.96–0.99)	< 0.001	1.09 (1.07–1.10)	< 0.001
70–79	1.06 (1.04–1.07)	< 0.001	1.22 (1.21–1.24)	< 0.001
≥ 80	1.27 (1.25–1.30)	< 0.001	1.32 (1.30–1.35)	< 0.001
**Sex**				
Female	Reference	—	Reference	—
Male	1.24 (1.22–1.25)	< 0.001	1.18 (1.17–1.19)	< 0.001
**Race**				
White	Reference	—	Reference	—
Black	1.15 (1.13–1.17)	< 0.001	0.99 (0.97–1.00)	0.091
Others^a^	0.89 (0.87–0.91)	< 0.001	0.77 (0.75–0.79)	< 0.001
Unknown	0.62 (0.54–0.72)	< 0.001	0.54 (0.47–0.62)	< 0.001
**Year of diagnosis**				
2000–2004	Reference	—	Reference	—
2005–2009	0.88 (0.87–0.90)	< 0.001	0.88 (0.87–0.89)	< 0.001
2010–2014	0.81 (0.80–0.82)	< 0.001	0.79 (0.78–0.80)	< 0.001
**Marital status**				
Married	Reference	—	Reference	—
Not married^b^	1.12 (1.11–1.14)	< 0.001	1.09 (1.08–1.11)	< 0.001
Unknown	1.04 (1.01–1.07)	0.003	1.01 (0.98–1.04)	0.638
**Grade^c^**				
I	Reference	—	Reference	—
II	1.64 (1.59–1.70)	< 0.001	1.34 (1.29–1.38)	< 0.001
III-IV	2.35 (2.27–2.43)	< 0.001	1.53 (1.47–1.58)	< 0.001
Unknown	3.51 (3.39–3.63)	< 0.001	1.44 (139–1.49)	< 0.001
**Tumor size (cm)**				
≤3	Reference	—	Reference	—
3–5	1.67 (1.64–1.70)	< 0.001	1.23 (1.21–1.25)	< 0.001
5–7	2.20 (2.16–2.24)	< 0.001	1.42 (1.39–1.45)	< 0.001
> 7	2.69 (2.64–2.75)	< 0.001	1.61 (1.58–1.64)	< 0.001
Unknown	3.13 (3.08–3.18)	< 0.001	1.46 (1.43–1.48)	< 0.001
**SEER stage**				
Localized	Reference	—	Reference	—
Regional	2.24 (2.20–2.28)	< 0.001	1.65 (1.62–1.69)	< 0.001
Distant	5.95 (5.84–6.06)	< 0.001	3.13 (3.06–3.20)	< 0.001
Unknown	3.69 (3.55–3.83)	< 0.001	1.37 (1.31–1.43)	< 0.001
**Nodal status**				
No	Reference	—	Reference	—
Yes	2.43 (2.40–2.46)	< 0.001	1.29 (1.27–1.31)	< 0.001
Unknown	3.11 (3.05–3.17)	< 0.001	1.26 (1.23–1.28)	< 0.001
**Surgery**				
No	Reference	—	Reference	—
Yes	0.21 (0.20–0.21)	< 0.001	0.36 (0.35–0.36)	< 0.001
Unknown	0.88 (0.82–0.94)	< 0.001	0.89 (0.83–0.95)	< 0.001
**Radiation**				
No/Unknown	Reference	—	Reference	—
Yes	1.60 (1.58–1.62)	< 0.001	1.01 (1.00–1.02)	0.034
**Chemotherapy**				
No/Unknown	Reference	—	Reference	—
Yes	1.24 (1.23–1.25)	< 0.001	0.62 (0.61–0.63)	< 0.001

Abbreviations: ASC, adenosquamous carcinoma; ADC, adenocarcinoma; SCC, squamous cell carcinoma; SEER, Surveillance Epidemiology and End Results database; HR, hazard ratio; CI, confidence interval.

^a^Others included American Indian/Alaskan native, and Asian/Pacific islander.

^b^Not married included separated, single (never married), divorced, unmarried or domestic partner and widowed.

^c^Grade I is well-differentiated; Grade II is moderately differentiated; Grade III is poorly differentiated; Grade IV is undifferentiated.

### Stratified analysis according to SEER stage and surgery

**Figure 2:**
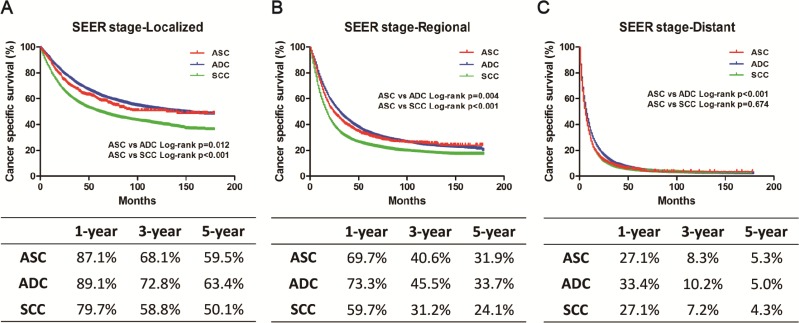
Kaplan-Meier plot and log-rank test for the cancer-specific survival (CSS) according to SEER stage among these three histological types (**A**) Survival curves in three groups with localized disease. ASC vs. ADC, *p* = 0.012; ASC vs. SCC, *p* < 0.001. (**B**) Survival curves in three groups with regional disease. ASC vs. ADC, *p* = 0.004; ASC vs. SCC, *p* < 0.001. (**C**) Survival curves in three groups with distant disease. ASC vs. ADC, *p* < 0.001; ASC vs. SCC, *p* = 0.674.

**Figure 3:**
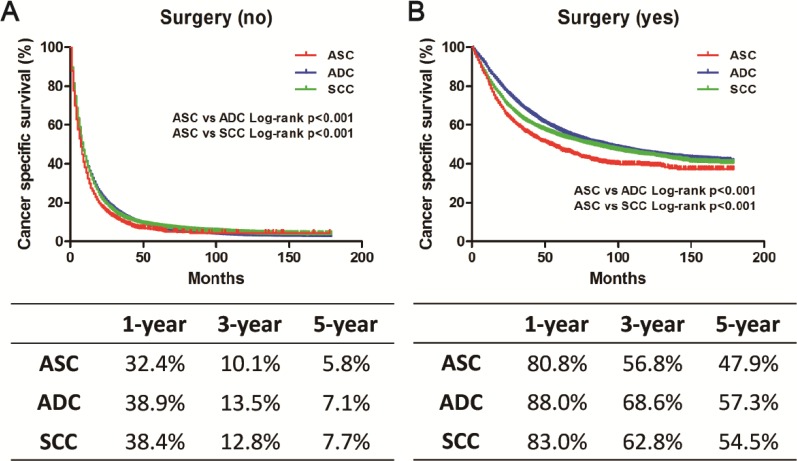
Kaplan-Meier plot and log-rank test for the cancer-specific survival (CSS) according to surgery among these three histological types (**A**) Survival curves in three groups without surgery treatment. ASC vs. ADC, *p* < 0.001; ASC vs. SCC, *p* < 0.001. (**B**) Survival curves in three groups with surgery treatment. ASC vs. ADC, *p* < 0.001; ASC vs. SCC, *p* < 0.001.

We stratified the study population according to the SEER stage or surgery to further evaluate the difference between ASC and ADC or SCC. The Kaplan-Meier method was used to evaluate CSS based on SEER stage or surgery (Figures 2–3). In the localized or regional subgroup, ASC patients were intermediate between ADC and SCC cases with regard to CSS. There was no significant difference in survival curves between ASC and SCC patients with distant disease. However, in the surgery group or non-surgery subgroup, ASC patients had worse survival compared with ADC or SCC patients. Univariate and multivariate Cox proportional hazard models were employed to evaluate the HRs of these three histology types for CSS, and the ASC histology type was set as the reference (Table 3). In the univariate and multivariate analysis, the ASC histology type was confirmed to be an independent risk factor for CSS in the surgery and non-surgery subgroup. However, different results were found in subgroups stratified according to SEER stage. In the univariate analysis, ASC patients had a worse prognosis compared with ADC patients. However, when compared with SCC patients, ASC patients had a better prognosis in terms of localized or regional disease, but there was no difference with regard to distant disease. In the multivariate analysis, ASC was an independent risk factor for CSS in the regional and distant subgroups, but no significant difference in the prognosis was found among these three histology types in the localized subgroup.

**Table 3:** Univariate and multivariate analysis of three histological types on cancer-specific survival (CSS) based on SEER stage or surgery

Characteristics	Univariate analysis		Multivariate analysis	
	HR (95% CI)	*p* value	HR (95% CI)	*p* value
**SEER stage**				
**Localized**				
ASC	Reference	—	Reference	—
ADC	0.87 (0.78–0.97)	0.012	0.90 (0.81–1.00)	.051
SCC	1.38 (1.23–1.53)	< 0.001	0.96 (0.86–1.07)	.430
**Regional**				
ASC	Reference	—	Reference	—
ADC	0.91 (0.85–0.97)	0.005	0.83 (0.77–0.88)	< 0.001
SCC	1.31 (1.22–1.40)	< 0.001	0.85 (0.80–0.91)	< 0.001
**Distant**				
ASC	Reference	—	Reference	—
ADC	0.89 (0.84–0.94)	< 0.001	0.89 (0.85–0.94)	< 0.001
SCC	1.01 (0.96–1.07)	0.656	0.88 (0.83–0.93)	< 0.001
**Surgery**				
**No**				
ASC	Reference	—	Reference	—
ADC	0.87 (0.83–0.91)	< 0.001	0.87 (0.83–0.91)	< 0.001
SCC	0.87 (0.83–0.91)	< 0.001	0.89 (0.85–0.94)	< 0.001
**Yes**				
ASC	Reference	—	Reference	—
ADC	0.72 (0.68–0.77)	< 0.001	0.87 (0.82–0.93)	< 0.001
SCC	0.83 (0.78–0.89)	< 0.001	0.81 (0.76–0.86)	< 0.001

Abbreviations: ASC, adenosquamous carcinoma; ADC, adenocarcinoma; SCC, squamous cell carcinoma; SEER, Surveillance Epidemiology and End Results database; HR, hazard ratio; CI, confidence interval.

### Survival analysis in matched groups

**Figure 4:**
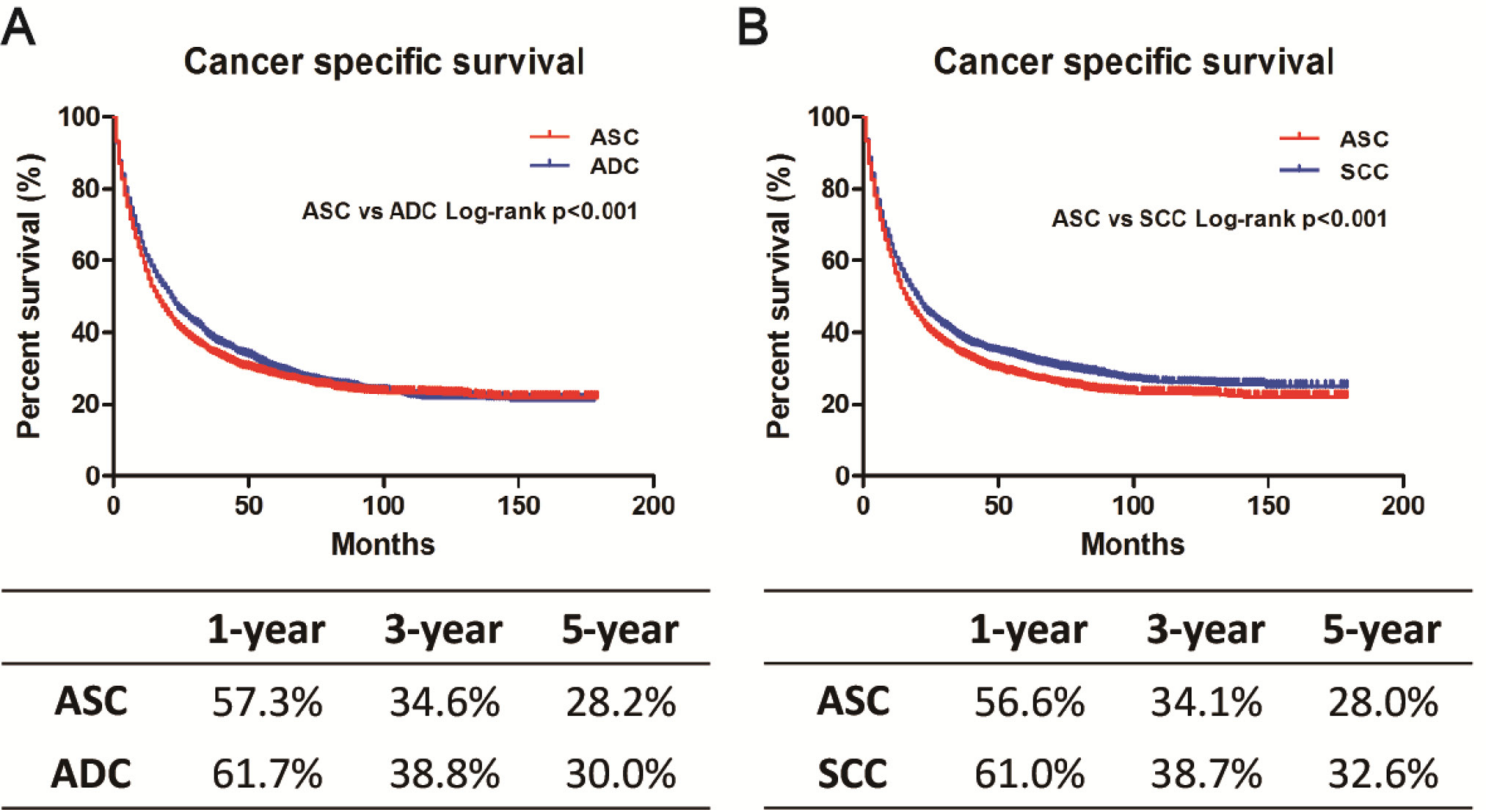
Kaplan-Meier plot and log-rank test for the cancer-specific survival (CSS) in 1:1 matched cohorts (**A**) Survival curves in 1:1 matched ASC and ADC. *p* < 0.001. (**B**) Survival curves in 1:1 matched ASC and SCC. *p* < 0.001.

To eliminate the confounding effect from an imbalance in baseline characteristics of these three patient groups on lung cancer outcomes, we conducted 1:1 matched case-control analysis using PSM to match ASC with ADC and SCC patients, respectively. We obtained a group of 8,490 patients (4,245 ASC and 4,245 ADC patients) and a group of 7,852 patients (3,926 ASC patients and 3,926 SCC patients) (Table 4A–4B). No significant differences in baseline characteristics were found between ASC and ADC or SCC patients in the matched groups. Interestingly, matched ASC patients exhibited a worse prognosis compared with matched ADC or matched SCC patients, respectively, using a Kaplan-Meier method (Figure 4). After adjustment for these covariates using multivariate analysis, ASC patients continued to have worse survival than ADC or SCC patients (ASC *vs.* ADC: HR = 1.14, 95% CI 1.08–1.20, *p* < 0.001; ASC *vs.* SCC: HR = 1.20, 95% CI 1.13–1.27, *p* < 0.001) (Supplementary Table 1A–1B).

**Table 4A:** The demographic and clinical characteristics of ASC and ADC patients in 1:1 matched group

Characteristics	ASC *N* = 4245 (%)	ADC *N* = 4245 (%)	*p* value^a^
**Age**			0.941
< 60	977 (23.0)	992 (23.4)	
60–69	1292 (30.4)	1304 (30.7)	
70–79	1413 (33.3)	1388 (32.7)	
≥ 80	563 (13.3)	561 (13.2)	
**Sex**			0.930
Female	1896 (44.7)	1900 (44.8)	
Male	2349 (55.3)	2345 (55.2)	
**Race**			0.603
White	3481 (82.0)	3526 (83.1)	
Black	471 (11.1)	447 (10.5)	
Others^b^	285 (6.7)	266 (6.3)	
Unknown	8 (0.2)	6 (0.1)	
**Year of diagnosis**			0.967
2000–2004	1192 (28.1)	1182 (27.8)	
2005–2009	1352 (31.8)	1353 (31.9)	
2010–2014	1701 (40.1)	1710 (40.3)	
**Marital status**			0.296
Married	2327 (54.8)	2396 (56.4)	
Not married^c^	1730 (40.8)	1675 (39.5)	
Unknown	188 (4.4)	174 (4.1)	
**Grade^d^**			0.318
I	42 (1.0)	55 (1.3)	
II	907 (21.4)	905 (21.3)	
III–IV	2079 (49.0)	2020 (47.6)	
Unknown	1217 (28.7)	1265 (29.8)	
**Tumor size (cm)**			0.783
≤3	1486 (35.0)	1509 (35.5)	
3–5	1164 (27.4)	1175 (27.7)	
5–7	627 (14.8)	595 (14.0)	
> 7	355 (8.4)	337 (7.9)	
Unknown	613 (14.4)	629 (14.8)	
**SEER stage**			0.821
Localized	985 (23.2)	986 (23.2)	
Regional	1572 (37.0)	1564 (36.8)	
Distant	1634 (38.5)	1650 (38.9)	
Unknown	54 (1.3)	45 (1.1)	
**Nodal status**			0.859
No	1861 (43.8)	1841 (43.4)	
Yes	2146 (50.6)	2157 (50.8)	
Unknown	238 (5.6)	247 (5.8)	
**Surgery**			0.224
No	2091 (49.3)	2104 (49.6)	
Yes	2138 (50.4)	2114 (49.8)	
Unknown	16 (0.4)	27 (0.6)	
**Radiation**			0.947
No/Unknown	2592 (61.1)	2595 (61.1)	
Yes	1653 (38.9)	1650 (38.9)	
**Chemotherapy**			0.645
No/Unknown	2424 (57.1)	2403 (56.6)	
Yes	1821 (42.9)	1842 (43.4)	

Abbreviations: ASC, adenosquamous carcinoma; ADC, adenocarcinoma; SCC, squamous cell carcinoma; SEER, Surveillance Epidemiology and End Results database.

^a^*p* value between ASC and ADC or SCC was calculated by chi-square test, respectively.

^b^Others included American Indian/Alaskan native, and Asian/Pacific islander.

^c^Not married included separated, single (never married), divorced, unmarried or domestic partner and widowed.

^d^Grade I is well-differentiated; Grade II is moderately differentiated; Grade III is poorly differentiated; Grade IV is undifferentiated.

**Table 4B:** The demographic and clinical characteristics of ASC and ASC patients in 1:1 matched group

Characteristics	ASC *N* = 3926	SCC *N* = 3926 (%)	*p* value^a^
**Age**			0.986
< 60	860 (21.9)	859 (21.9)	
60–69	1220 (31.1)	1212 (30.9)	
70–79	1341 (34.2)	1339 (34.1)	
≥ 80	505 (12.9)	516 (13.1)	
**Sex**			0.466
Female	1720 (43.8)	1688 (43.0)	
Male	2206 (56.2)	2238 (57.0)	
**Race**			0.052
White	3343 (85.2)	3299 (84.0)	
Black	403 (10.3)	399 (10.2)	
Others^b^	178 (4.5)	221 (5.6)	
Unknown	2 (0.1)	7 (0.2)	
**Year of diagnosis**			0.871
2000–2004	1080 (27.5)	1097 (27.9)	
2005–2009	1270 (32.3)	1251 (31.9)	
2010–2014	1576 (40.1)	1578 (40.2)	
**Marital status**			0.078
Married	2172 (55.3)	2162 (55.1)	
Not married^c^	1646 (41.9)	1621 (41.3)	
Unknown	108 (2.8)	143 (3.6)	
**Grade^d^**			0.881
I	36 (0.9)	39 (1.0)	
II	875 (22.3)	861 (21.9)	
III-IV	1907 (48.6)	1938 (49.4)	
Unknown	1108 (28.2)	1088 (27.7)	
**Tumor size (cm)**			0.878
≤3	1333 (34.0)	1341 (34.2)	
3–5	1111 (28.3)	1075 (27.4)	
5–7	585 (14.9)	583 (14.8)	
> 7	333 (8.5)	338 (8.6)	
Unknown	564 (14.4)	589 (15.0)	
**SEER stage**			0.798
Localized	954 (24.3)	938 (23.9)	
Regional	1457 (37.1)	1443 (36.8)	
Distant	1464 (37.3)	1500 (38.2)	
Unknown	51 (1.3)	45 (1.1)	
**Nodal status**			0.668
No	1732 (44.1)	1715 (43.7)	
Yes	2003 (51.0)	2003 (51.0)	
Unknown	191 (4.9)	208 (5.3)	
**Surgery**			0.650
No	2044 (52.1)	2023 (51.5)	
Yes	1873 (47.7)	1897 (48.3)	
Unknown	9 (0.2)	6 (0.2)	
**Radiation**			0.908
No/Unknown	2401 (61.2)	2396 (61.0)	
Yes	1525 (38.8)	1530 (39.0)	
**Chemotherapy**			0.326
No/Unknown	2283 (58.2)	2240 (57.1)	
Yes	1643 (41.8)	1686 (42.9)	

Abbreviations: ASC, adenosquamous carcinoma; ADC, adenocarcinoma; SCC, squamous cell carcinoma; SEER, Surveillance Epidemiology and End Results database.

^a^*p* value between ASC and ADC or SCC was calculated by chi-square test, respectively.

^b^Others included American Indian/Alaskan native, and Asian/Pacific islander.

^c^Not married included separated, single (never married), divorced, unmarried or domestic partner and widowed.

^d^Grade I is well-differentiated; Grade II is moderately differentiated; Grade III is poorly differentiated; Grade IV is undifferentiated.

### Subgroup analysis in matched groups

**Figure 5:**
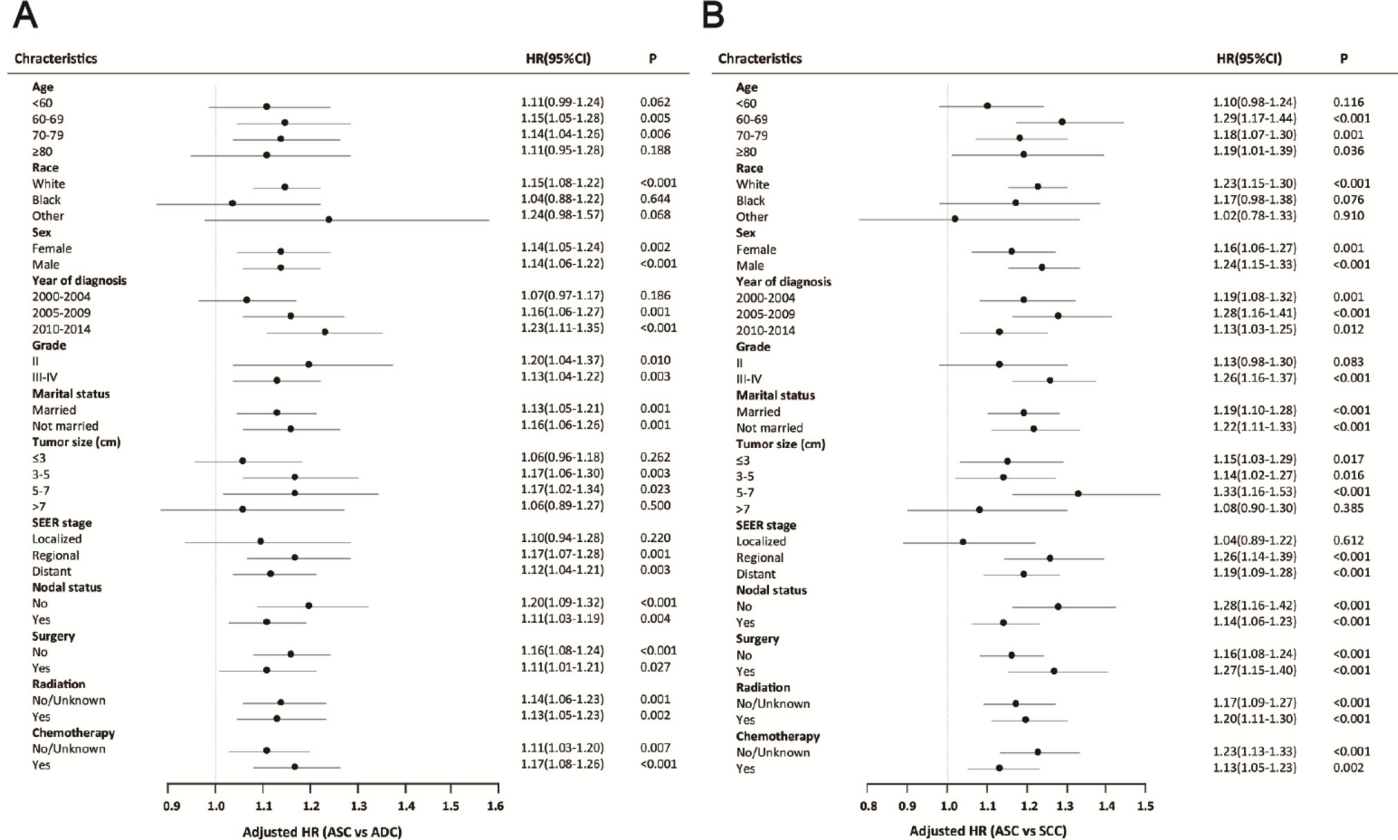
Forest plot of hazard ratios (HRs) for ASC versus ADC or SCC in the subgroup analysis The circle and line segment represent the HR and 95% confident interval of each subgroup. An HR > 1.00 indicates higher risk for CSS in patients with ASC compared with ADC or SCC and vice versa.

To further elucidate the characteristics of lung ASC patients, we undertook subgroup analysis in matched groups (Figure 5). Analysis for most of subgroups showed that ASC patients had a worse prognosis than ADC or SCC patients. However, in the subgroup analysis of ASC and ADC patients, there was no significant difference for HRs for age < 60 (HR = 1.11, 95% CI 0.99–1.24, *p* = 0.062), age ≥ 80 (HR = 1.11, 95% CI 0.95–1.28, *p* = 0.188), black race (HR = 1.04, 95% CI 0.88–1.22, *p* = 0.644), other races (HR = 1.24, 95% CI 0.98–1.57, *p* = 0.068), year of diagnosis between 2000 and 2004 (HR = 1.07, 95% CI 0.97–1.17, *p* = 0.186), tumor size ≤ 3 cm (HR = 1.06, 95% CI 0.96–1.18, *p* = 0.262), tumor size > 7 cm (HR = 1.06, 95% CI 0.89–1.27, *p* = 0.500), and localized subgroup (HR = 1.10, 95% CI 0.94–1.28, *p* = 0.220). In the subgroup analysis of ASC and SCC patients, no significant difference was found for HRs for age < 60 (HR = 1.10, 95% CI 0.98–1.24, *p* = 0.116), black race (HR = 1.17, 95% CI 0.98–1.38, *p* = 0.076), other races (HR = 1.02, 95% CI 0.78–1.33, *p* = 0.910), Grade II (HR = 1.13, 95% CI 0.98–1.30, *p* = 0.083), tumor size > 7 cm (HR = 1.08, 95% CI 0.90–1.30, *p* = 0.385), and localized subgroup (HR = 1.04, 95% CI 0.89–1.22, *p* = 0.612).

## DISCUSSION

The present study identified 4,245 ASC patients from the SEER database and this was the largest number of patients identified compared with other studies. Through comparative analysis, we found that ASC patients shared different demographic and clinical characteristics from ADC and SCC patients. ASC patients carried a better prognosis than ADC and SCC patients. Interestingly, after 1:1 matching ASC with ADC or SCC using PSM, ASC patients had a worse prognosis than ADC and SCC patients. Furthermore, multivariate and subgroup analysis supported the ASC histology type to be an independent risk factor for the prognosis compared with ADC and SCC histology types.

The demographic and clinical characteristics of ASC were varying and inconsistent in current studies. With regard to demographic characteristics, Cooke et al. [8] analyzed early data from the SEER database (1998–2002) and showed that age and the male/female ratio of ASC lay between that of ADC and SCC. Fewer ASC patients were white race compared with ADC and SCC patients. In a relatively large study undertaken in Japan by Maeda and colleagues, the mean age of 114 enrolled ASC patients was lower than that of ADC and SCC patients. The male/female ratio of ASC patients was higher than that of ADC patients, but lower than that of SCC patients [5]. A French study showed that, in 141 ASC patients from two centers, age, male/female ratio and percentage of smoking were similar to those in ADC patients, but were distinctly lower than those in SCC patients [4]. A retrospective study carried out in China evaluated 72 ASC patients, and found 63.7% of them to be male with a median age of 60 years, and that 55.6% had smoking history [13]. In another study that identified 127 ASC patients, the male/female ratio and percentage of smokers were even higher to 72% and 90%, respectively [14]. Our results were closer to those of the study by Cooke and colleagues. In our study, the age, male/female ratio and race of ASC patients were intermediate between ADC and SCC patients. The reason for such obvious differences between our study and those of others may lie in small study cohorts or misclassification of patients for other studies. Besides, we included, for the first time, the marital status for ASC patients. We found a similar proportion of married/unmarried in ASC patients with that in ADC patients, but more SCC patients were unmarried.

With regard to the clinical characteristics, Cooke et al. [8] showed that the tumor grade of ASC patients was higher than that of ADC and SCC patients. The tumor size and prevalence of nodal metastasis for ASC patients were intermediate between ADC and SCC. Filosso et al. [6] concluded, from a small number of ASC patients, that the tumor stage was higher in ASC patients compared with ADC and SCC cases. Similarly, Ruffini et al. [15] and Cakir et al. [16] concluded that ASC presented at a more advanced stage than ADC and SCC. In the study by Maeda and colleagues, ASC patients had a lower tumor grade, a larger tumor size, and higher proportions of nodal metastasis and stage IIIA compared with ADC patients, but there was no significant difference between ASC and SCC patients. Besides, the proportion of patients who underwent chemotherapy in these three groups was not significantly different [5]. Mordant et al. [4] showed that ASC had the largest tumor size among these three groups. In the present study, ASC patients had a higher tumor grade than ADC and SCC patients, which was consistent with those of Cooke et al. but different to those of Maeda and coworkers. In terms of tumor size, ASC lay between ADC and SCC in our study. Interestingly, more ASC patients were diagnosed at an early stage, compared with ADC and SCC cases. The proportion of nodal metastasis in ASC patients was slightly lower than that in ADC and SCC patients. With regard to treatment (surgery, radiation and chemotherapy), we evaluated ASC in a large population for the first time. We found that more ASC patients underwent surgery, compared with ADC and SCC patients. The proportion of patients who underwent radiation treatment for ASC was lower than that for SSC, and the proportion receiving chemotherapy was lower for ASC than that for ADC.

Most studies have suggested that ASC patients had worse survival than ADC and ASC patients [5–8, 17]. However, Uramoto et al. [9] showed that the prognosis of ASC patients was similar to that of ADC and ASC patients, which was in accordance with the work of Hsia et al. [10]. Interestingly, we found that the prognosis of ASC was better than that of ADC and SCC, but stratified analysis did not support this result and showed that survival for ASC patients was worse than that for ADC and SCC in the surgery and non-surgery subgroup. Multivariate analysis showed that ASC was an independent risk factor for the prognosis. Considering the effect of confounding factors on survival outcomes, we originally used a PSM to 1:1 match ASC with ADC or SCC. As expected, ASC patients had worse survival compared with ADC or SCC cases. The same result was found in multivariate analysis for matched groups. In subgroup analysis, ASC was identified to be an independent factor for a poor prognosis in most subgroups. Moreover, CSS at 1, 3 and 5 years for unmatched or matched ASC patients were obviously lower than that reported previously [8]. This contradiction may have been caused by early-stage disease and surgery treatment in ASC patients enrolled in most previous studies. We further confirmed that stage and surgery treatment were significantly associated with the survival of ASC patients, and were identified independent risk factors for the prognosis in univariate and multivariate analysis.

The current treatment strategy for ASC is limited and has developed mainly from studies on ADC and SCC. The prevalence of the epidermal growth factor receptor (EGFR) mutation in ASC has been reported to affect 15.4% to 44.0% of patients, which was calculated from a limited number of cases [18–21]. However, EGFR-tyrosine kinase inhibitors have been demonstrated to be effective treatment for ASC patients with the EGFR mutation [22]. In the present study, data on the EGFR mutation were not provided by the SEER database, but other significant findings were identified to assist with clinical decision for ASC treatment. In the multivariate analysis, surgery and chemotherapy, but not radiation, were protective factors for the prognosis in ASC patients. Surgery treatment for ASC patients could obviously improve survival, but the prognosis was still worse than that for ADC and SCC patients in surgery subgroup. Additionally, subgroup analysis showed that ASC contributed to worse survival compared with ADC or SCC. Thus, it reminded clinical physicians to take a more positive treatment strategy for ASC patients.

Besides, the present study had several limitations. Firstly, data on smoking history, self-reported information from patients, laboratory tests and imaging were not provided by the SEER database. Secondly, AJCC stage and metastasis site were excluded due to its inadequate information. Additionally, the detailed chemotherapy regimens and information for targeted drug were not provided by SEER database. The lack of these information may affect the survival analysis. Lastly, gene mutation data of patients (including those of the EGFR, KRAS, and BRAF mutation) were not provided by the SEER database which reduced the importance of our investigation in terms of clinical application [23].

In summary, we found ASC to be a unique histological type in lung cancer. It was intermediate between ADC and SCC with regard to age, sex, race, year of diagnosis, tumor size and SEER stage. Compared with ADC and SCC, ASC patients presented with a higher tumor grade and lower prevalence of nodal metastasis. More ASC patients underwent surgery and a lower proportion underwent radiation treatment and chemotherapy. The prognosis of ASC patients was worse than that of ADC and SCC patients after adjustment for baseline characteristics. Multivariate and subgroup analysis supported the notion that the ASC histology type was an independent factor for a poor prognosis. These results from a large cohort of ASC patients provided a deep insight to ASC and contributed to diagnosis and treatments of ASC for clinical physicians.

## SUPPLEMENTARY MATERIALS TABLE


