# Supplementary material for: Clinicopathological characteristics and survival outcomes in adenosquamous carcinoma of the lung: a population-based study from the SEER database
Source: Oncotarget. 2017 Dec 21;9(8):8133–46. doi: 10.18632/oncotarget.23550 (PMC5814288; doi:10.18632/oncotarget.23550)
Supplement: Supplementary file 1 [file oncotarget-09-8133-s001.pdf]

Clinicopathological characteristics and survival outcomes in adenosquamous carcinoma of the lung: a population-based study from the SEER database

SUPPLEMENTARY MATERIALS

Supplementary Table 1A: Multivariate analysis of cancer-specific survival (CSS) between ASC and ADC

Characteristics	Multivariate analysis	
	HR (95%CI)	*p* value
**Histological type**		
ADC	Reference	—
ASC	1.14 (1.08–1.20)	< 0.001
**Age**		
< 60	Reference	—
60–69	1.15 (1.06–1.24)	< 0.001
70–79	1.23 (1.23–1.43)	< 0.001
≥ 80	1.42 (1.29–1.56)	< 0.001
**Sex**		
Female	Reference	—
Male	1.21 (1.14–1.28)	< 0.001
**Race**		
White	Reference	—
Black	1.02 (0.94–1.11)	0.659
Others[a]	0.72 (0.64–0.80)	< 0.001
Unknown	0.59 (0.30–1.13)	0.111
**Year of diagnosis**		
2000–2004	Reference	—
2005–2009	0.93 (0.87–1.00)	0.049
2010–2014	0.78 (0.73–0.84)	< 0.001
**Marital status**		
Married	Reference	—
Not married[b]	1.08 (1.02–1.15)	0.009
Unknown	1.08 (0.95–1.24)	0.247
**Grade[c]**		
I	Reference	—
II	1.07 (0.79–1.45)	0.647
III-IV	1.31 (0.97–1.76)	0.077
Unknown	1.18 (0.87–1.59)	0.286
**Tumor size (cm)**		
≤3	Reference	—
3–5	1.29 (1.20–1.39)	< 0.001
5–7	1.55 (1.42–1.70)	< 0.001
> 7	1.70 (1.53–1.89)	< 0.001
Unknown	1.60 (1.47–1.75)	< 0.001
**SEER stage**		
Localized	Reference	—
Regional	1.80 (1.63–2.00)	< 0.001
Distant	3.72 (3.33–4.16)	< 0.001
Unknown	1.36 (1.04–1.77)	0.024
**Nodal status**		
No	Reference	—
Yes	1.29 (1.20–1.39)	< 0.001
Unknown	1.49 (1.32–1.69)	< 0.001
**Surgery**		
No	Reference	—
Yes	0.43 (0.39–0.47)	< 0.001
Unknown	0.74 (0.52–1.06)	0.103
**Radiation**		
No/ Unknown	Reference	—
Yes	1.11 (1.05–1.19)	< 0.001
**Chemotherapy**		
No/ Unknown	Reference	—
Yes	0.63 (0.59–0.67)	< 0.001

Abbreviations: ASC, adenosquamous carcinoma; ADC, adenocarcinoma; SCC, squamous cell carcinoma; SEER, Surveillance Epidemiology and End Results database; HR, hazard ratio; CI, confidence interval.

[a] Others included American Indian/Alaskan native, and Asian/Pacific islander.

[b] Not married included separated, single (never married), divorced, unmarried or domestic partner and widowed.

[c] Grade I is well-differentiated; Grade II is moderately differentiated; Grade III is poorly differentiated; Grade IV is undifferentiated.

Supplementary Table 1B: Multivariate analysis of cancer-specific survival (CSS) between ASC and SCC

Characteristics	Multivariate analysis	
	HR (95%CI)	*p* value
**Histological type**		
SCC	Reference	—
ASC	1.20 (1.13–1.27)	< 0.001
**Age**		
< 60	Reference	—
60–69	1.10 (1.01–1.19)	0.022
70–79	1.31 (1.21–1.42)	< 0.001
≥ 80	1.33 (1.20–1.47)	< 0.001
**Sex**		
Female	Reference	—
Male	1.16 (1.10–1.23)	< 0.001
**Race**		
White	Reference	—
Black	1.01 (0.92–1.11)	0.836
Others[a]	0.79 (0.69–0.90)	< 0.001
Unknown	0.61 (0.27–1.37)	0.232
**Year of diagnosis**		
2000–2004	Reference	—
2005–2009	0.96 (0.89–1.03)	0.263
2010–2014	0.84 (0.78–0.91)	< 0.001
**Marital status**		
Married	Reference	—
Not married[b]	1.09 (1.03–1.16)	0.005
Unknown	0.91 (0.77–1.08)	0.273
**Grade[c]**		
I	Reference	—
II	1.07 (0.76–1.52)	0.686
III–IV	1.18 (0.84–1.67)	0.34
Unknown	1.10 (0.78–1.56)	0.585
**Tumor size (cm)**		
≤ 3	Reference	—
3–5	1.33 (1.22–1.44)	< 0.001
5–7	1.53 (1.39–1.68)	< 0.001
> 7	1.72 (1.54–1.92)	< 0.001
Unknown	1.56 (1.42–1.71)	< 0.001
**SEER stage**		
Localized	Reference	—
Regional	1.64 (1.48–1.83)	< 0.001
Distant	3.10 (2.75–3.49)	< 0.001
Unknown	1.34 (1.01–1.78)	0.041
**Nodal status**		
No	Reference	—
Yes	1.28 (1.19–1.39)	< 0.001
Unknown	1.46 (1.27–1.68)	< 0.001
**Surgery**		
No	Reference	—
Yes	0.36 (0.33–0.39)	< 0.001
Unknown	0.85 (0.47–1.54)	0.585
**Radiation**		
No/ Unknown	Reference	—
Yes	1.05 (0.98–1.12)	0.169
**Chemotherapy**		
No/ Unknown	Reference	—
Yes	0.65 (0.61–0.70)	< 0.001

Abbreviations: ASC, adenosquamous carcinoma; ADC, adenocarcinoma; SCC, squamous cell carcinoma; SEER, Surveillance Epidemiology and End Results database; HR, hazard ratio; CI, confidence interval.

[a] Others included American Indian/Alaskan native, and Asian/Pacific islander.

[b] Not married included separated, single (never married), divorced, unmarried or domestic partner and widowed.

[c] Grade I is well-differentiated; Grade II is moderately differentiated; Grade III is poorly differentiated; Grade IV is undifferentiated.
